# A novel gammapolyomavirus in a great cormorant (*Phalacrocorax carbo*)

**DOI:** 10.1007/s00705-022-05478-8

**Published:** 2022-05-28

**Authors:** Enikő Fehér, Eszter Kaszab, Krisztina Bali, Márton Hoitsy, Endre Sós, Krisztián Bányai

**Affiliations:** 1 Veterinary Medical Research Institute, Hungária krt 21, 1143 Budapest, Hungary; 2 Conservation and Veterinary Services, Budapest Zoo and Botanical Garden, Állatkerti krt. 6-12, 1164 Budapest, Hungary; 3 University of Veterinary Medicine, István utca 2, 1078 Budapest, Hungary

## Abstract

In this study, the complete genome of a novel polyomavirus detected in a great cormorant (*Phalacrocorax carbo*) was characterized. The 5133-bp-long genome of the cormorant polyomavirus has a genomic structure typical of members of the genus *Gammapolyomavirus*, family *Polyomaviridae*, containing open reading frames encoding the large and small tumor antigens, viral proteins 1, 2, and 3, and the X protein. The large tumor antigen of the cormorant polyomavirus shares 45.6–50.4% amino acid sequence identity with the homologous sequences of other gammapolyomaviruses. These data, together with results of phylogenetic analysis, suggest that this cormorant polyomavirus should be considered the first member of a new species within the genus *Gammapolyomavirus*, for which we propose the name “*Phalacrocorax carbo polyomavirus 1*”.

## Introduction

Members of the family *Polyomaviridae* infect mammals, birds, and fish [1–3]. The biology of avian and mammalian polyomaviruses differs significantly. Avian polyomaviruses are not highly species-specific, and they cause acute, fulminant, and often fatal disease in their susceptible hosts. In contrast, mammalian polyomaviruses show rigorous host species specificity and typically cause inapparent infections in immunocompetent hosts [2]. The family *Polyomaviridae* comprises eight genera. Viruses of the genera *Alpha*-, *Beta*-, and *Deltapolyomavirus*, as well as the recently established genera *Epsilon*- and *Zetapolyomavirus* have been detected from mammals, whereas members of the genus *Gammapolyomavirus* infect birds. Polyomaviruses of fish have been assigned to the new genera *Etapolyomavirus* and *Thetapolyomavirus* [1].

Polyomaviruses are characterized by nonenveloped icosahedral particles, 40–45 nm in diameter, that enclose a circular dsDNA genome of 3962–7369 bp [2]. The polyomavirus genes are expressed in a time-dependent manner. The products of the early genes are primarily regulatory proteins (e.g., the large and small tumor antigens [LTA and STA]), and the late genes code for the viral proteins (VPs) VP1, VP2, and VP3, which are responsible for virion formation [2, 4–6]. Additional coding capacity has been noted; for example, the genome of avian polyomaviruses may encode an X protein or a VP4 protein downstream of the replication origin [2, 5–8].

Our knowledge about the genetic diversity of mammalian polyomaviruses has increased rapidly over the past 10 years, which has led to an extended taxonomic classification, with more than 100 species. Gammapolyomaviruses are represented by nine species [1–3, 7]. Goose hemorrhagic polyomavirus and budgerigar fledgling disease virus, two well-characterized, high-mortality avian polyomaviruses, have been described in a number of bird species that might play a role in their natural circulation [8, 9]. Hence, it seems plausible that wild birds serve as hosts for novel gammapolyomaviruses as well as for some highly pathogenic gammapolyomaviruses associated with economic losses.

In this study, wild birds that died in 2019 at the Zoo and Botanical Garden, Budapest (Hungary), were tested for polyomaviruses. Approximately 50-100 mg of internal organ tissue samples were homogenized in PBS using a TissueLyzer LT instrument (QIAGEN, Hilden, Germany) and were centrifuged for 10,000 × *g* for 5 min. Nucleic acid was extracted using a ZiXpress-32^®^ Automated Nucleic Acid Purification Instrument and a ZiXpress-32^®^ Viral Nucleic Acid Extraction Kit (Zinexts Life Science Corp., New Taipei City, Taiwan) from a mixture of the prepared samples from each bird.

Polyomavirus DNA was detected using a broad-spectrum nested PCR assay with the primer sets VP1-1f and VP1-1r, and VP1-2f and VP1-2r, described by Johne and co-workers [10]. Sequencing of the amplicons revealed traces of polyomavirus sequence in one out of 32 specimens collected from kidney and liver samples from a great cormorant (*Phalacrocorax carbo*). The bird was admitted to the zoo’s rescue station with presumed traumatic injuries, but detailed pathological findings were not available.

The back-to-back PCR primers PyV_20190702-2_F (5’-TGGGAAGATGTACTATAGGGGTCTTC-3’) and PyV_20190702-2_R (5’-TCTGACTGCACAACAAACCCAC-3’) (annealing at 65 °C) were designed for amplification of the circular polyomavirus genome using Phusion DNA polymerase according to the manufacturer’s instructions (Thermo Fisher Scientific, Waltham, MA, USA). The PCR product was purified and prepared for next-generation sequencing using an Illumina NextSeq™ 500 platform as described elsewhere [11]. Sequence contigs were obtained by *de novo* assembly using Geneious Prime^®^ v.2020.2.4 (Biomatters, Auckland, New Zealand) and CLC Genomics Workbench v9 (QIAGEN, Hilden, Germany) software. The ORFs were predicted using the Open Reading Frame Finder tool (https://www.ncbi.nlm.nih.gov/orffinder/). The sequences were edited using AliView software and were aligned using the MAFFT algorithm implemented in the Geneious Prime software [12]. Maximum-likelihood phylogenetic analysis was performed using PhyML software (LG+G+I+F model, aLRT SH-like branch support) using reference sequences [1, 13]. The phylogenetic tree was visualized and edited using MEGA6 software [14].

**Fig. 1:**
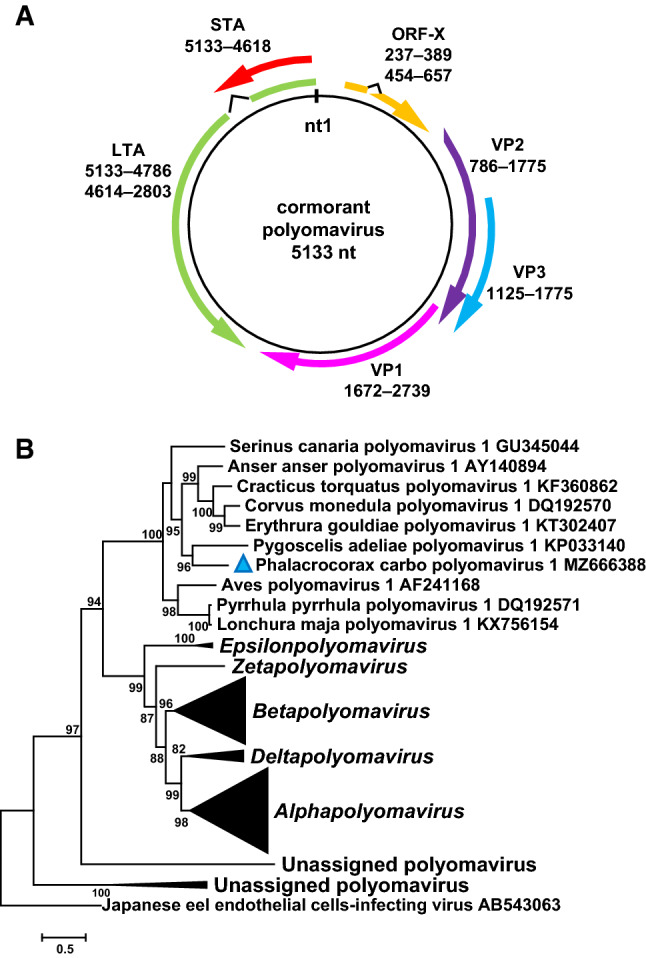
(A) Schematic representation of the genomic structure of cormorant polyomavirus. (B) Maximum-likelihood phylogenetic tree of LTA aa sequences of polyomaviruses constructed using PhyML software, applying the GTR+G+I model and aLRT SH-like branch support. Branches with < 80 support were hidden. The sequence of Japanese eel endothelial cells-infecting virus (GenBank accession number AB543063) was used to root the tree. The cormorant polyomavirus (*Phalacrocorax carbo polyomavirus 1*) is indicated by a blue triangle

Altogether, 528,035 sequence reads mapped to the novel genome with a sequencing depth of > 3100. The priming site of the back-to-back PCR was determined by Sanger sequencing, resulting in lower sequencing depth in this region. The novel genome (GenBank accession number MZ666388) was found to be 5133 bp long, and the genomic structure resembles that of gammapolyomaviruses, containing the putative ORFs encoding the LTA, STA, VP1, VP2, and VP3 proteins (Fig. 1, Table 1) [1–8]. Furthermore, ORF-X was predicted upstream of the VP2. We observed signatures of mRNA splicing in both the LTA and ORF-X genes (Fig. 1, Table 1). Analysis of representative complete genome sequences of members of all polyomavirus species, performed using RDP4 software, did not reveal any recombination events affecting the genome of the cormorant polyomavirus (CoPyV) [15].

**Table 1:** Main characteristics of the cormorant polyomavirus genome described in this study. Locations of the ORFs were determined using the first nucleotide upstream of the large/small tumor antigen ORFs as position 1

	Location (nt)	Length		Sequence identity (%) to other gammapolyomaviruses (nt/aa)
		ORF (nt)	Protein (aa)	
LTA	5133–4786, 4614–2803	2160	719	48.8–54.1/45.6–50.4
STA	5133–4618	516	171	34.8–47.5/27.8–41.3
VP1	1672–2739	1068	355	53.2–62.5/52.9–66.6
VP2	786–1775	990	329	25.7–36.4/37.8–46.8
VP3	1125–1775	651	216	38.7–47.8/27.6–35.8
ORF-X	237–389, 454–657	357	118	17.9–33.3/7.6–26.0

Typical motifs similar to those in other avian polyomaviruses could be identified in the LTA of CoPyV, including the polyomavirus conserved region (CR1, LEELL), the hexapeptide in the J domain (HPDKGG), the pRB1-binding motif (LHAEE), the nuclear localization signal (NLS, TPPKDRAT), the zinc finger motif (CETCKAQKKDMPFRMLKRKWVGGHIDDH), and the ATPase motifs (GGVNTGKT and GAVPVNLE) [4, 6, 7]. The VP3 started with an in-frame methionine of the VP2, as part of the motif MALMPY, which conformed to the consensus motif MALXXΦ (Φ = W, F, Y) described also for other polyomaviruses [4]. The C-terminal region of the putative VP2 and VP3 proteins of CoPyV and all other gammapolyomaviruses is rich in arginine (R) and lysine (K), which may be components of functional NLSs [4]. Although NLSs have been recognized in VP1 proteins of mammalian polyomaviruses [4], an accumulation of basic amino acids is not typical for this region of avian polyomaviruses.

In the LTA-based phylogenetic tree, the CoPyV sequence branched together with gammapolyomavirus sequences (Fig. 1). Each of the main coding sequence of CoPyV and the gammapolyomaviruses shared a maximum of 62.5% nt and 66.6% aa sequence identity in pairwise comparisons, showing the highest values with sequences from goose hemorrhagic polyomavirus, Adélie penguin polyomavirus, and butcherbird polyomavirus. According to the demarcation criteria for polyomaviruses, including a genetic distance of > 15% for the LTA aa sequence [3], CoPyV may be the first member of a novel species within the genus *Gammapolyomavirus*, for which we propose the name “*Phalacrocorax carbo polyomavirus 1*”.

## Data Availability

The sequence data are available in the GenBank database with accession number MZ666388.
